# Fieldable genotyping of *Bacillus anthracis *and *Yersinia pestis *based on 25-loci Multi Locus VNTR Analysis

**DOI:** 10.1186/1471-2180-8-21

**Published:** 2008-01-29

**Authors:** Andrea Ciammaruconi, Saverio Grassi, Riccardo De Santis, Giovanni Faggioni, Valentina Pittiglio, Raffaele D'Amelio, Alessandra Carattoli, Antonio Cassone, Gilles Vergnaud, Florigio Lista

**Affiliations:** 1 Histology and Molecular Biology Section, Army Medical Research Center, Via Santo Stefano Rotondo 4, 00184 Rome, Italy; 2 Direzione Generale della Sanità Militare, Via Santo Stefano Rotondo 4, 00184 Rome, Italy; 3 Dipartimento di Scienze Mediche, II Facoltà di Medicina e Chirurgia Università "La Sapienza", Via di Grottarossa 1039, 00189 Rome, Italy; 4 Division of Analytical Microbiology, Centre d'Etudes du Bouchet, BP3, 91710 Vert le Petit (France); 5 Institut de Génétique et Microbiologie, Univ Paris-Sud Orsay, F-91405, France; CNRS, Orsay, F-91405, France; 6 Department of Infectious, Parasitic, and Immunomediated Diseases, Istituto Superiore di Sanità, Viale Regina Elena, 299, 00161 Rome, Italy

## Abstract

**Background:**

Anthrax and plague are diseases caused by *Bacillus anthracis *and *Yersinia pestis *respectively. These bacteria are etiological agents for worldwide zoonotic diseases and are considered among the most feared potential bioterror agents. Strain differentiation is difficult for these microorganisms because of their high intraspecies genome homogeneity. Moreover, fast strain identification and comparison with known genotypes may be crucial for naturally occurring outbreaks versus bioterrorist events discrimination.

**Results:**

Thirty-nine *B. anthracis *and ten *Y. pestis *strains, representative of the species genetic diversity, were genotyped by Agilent 2100 Bioanalyzer using previously described Multiple Locus VNTR Analysis assays (MLVA). Results were compared to previous data obtained by standard genotyping system (capillary electrophoresis on automatic sequencer) and, when necessary, direct amplicon sequencing. A reference comparison table containing actual fragment sizes, sequencer sizes and Agilent sizes was produced.

**Conclusion:**

In this report an automated DNA electrophoresis apparatus which provides a cheaper alternative compared to capillary electrophoresis approaches was applied for genotyping of *B. anthracis *and *Y. pesti*s. This equipment, uses pre-cast gels and provides easy transportation, low maintenance and overall general logistic requirements and costs, is easy to set up and provides rapid analysis. This platform is a candidate for on-site MLVA genotyping of biothreat agents as well as other bacterial pathogens. It is an alternative to the more expensive and demanding capillary electrophoresis methods, and to the less expensive but more time-consuming classical gel electrophoresis approach.

## Background

*Bacillus anthracis *is a Gram-positive spore-forming bacillus that causes anthrax [1,2]. This bacterium is commonly found in soil and is responsible for diseases of herbivores and other mammals including humans. Anthrax is still endemic in many countries, Middle East, Africa, North, Central and South America, as well as other areas of the world [3]. The site of entry determines different forms of anthrax, cutaneous, gastrointestinal, and inhalation; the latter form is highly fatal, with a mortality rate of up to 80% in the absence of an adequate antimicrobial therapy.

*Yersinia pestis *is a Gram-negative bacterium, etiological agent of plague. The bacterium is transmitted by fleas or aerosols, causing different forms of plague: bubonic, septicemic or pneumonic [4,5]. *Y*.*pestis *is often associated with the wellknown Black Death plague of the Middle Ages, a pandemic that had killed a third of European population in the 14th and 15th centuries, but approximately 2,000 human cases still occur worldwide each year [5]. Primary pneumonic plague is rapidly progressive and virulent, and, as inhalation anthrax, with a mortality rate close to 100% in the absence of a timely treatment. *Y. pestis *and *B. anthracis *are both considered serious threats and potential bioterrorism agents [6] because of their evaluation as bioweapons by Soviet Union and United States laboratories during the past decades. Above all, *B. anthracis *gained renewed attention in 2001, when letters containing anthrax spores were mailed causing the death of five persons and infecting 17 others, while probably hundreds of people were exposed to infectious spores [7]. Both agents are classified by the US Centre for Disease Control and Prevention in the Bioterrorism Disease Agent List as Category A microrganism, the most dangerous ones, because of easy dissemination and transmission, high mortality and impact to public health.

*B. anthracis *and *Y. pestis *both show very low intraspecies genetic diversity [8-10], making very challenging the rapid and accurate differentiation among individual biovars and strains. Nevertheless, finding a way to differenziate the strains by molecular genotyping, remains essential for discrimination between naturally occurring versus intentional outbreaks. The importance of forensic microbiology, as this field is know called, was demonstrated during the 2001 events, and previously by Tokyo [11] and Sverdlovsk [12] incidents. Finally genetic characterization of isolates allows to increase information about worldwide bacterial distribution and epidemiology.

Standard genotyping methods require either highly discriminative but heavy, and relatively expensive devices such as automated capillary electrophoresis devices, or cheaper, easy to use but more time consuming and with lower resolution power such as agarose gels (for a review of bacterial MLVA genotyping see [13]). A new miniaturized platform for quantification and separation of nucleic acid molecules, Agilent 2100 Bioanalyzer, has shown accuracy, precision and high feasibility along with speed and moderate cost reagents. This platform is based on microfluidic technology and allows to analyze 12 DNA samples in 30 minutes. The device, also called "Lab on a Chip", integrates multiple functions onto a single apparatus, so that sample dispensing, separation, detection and analysis are performed on the same support (a 5 × 5 cm chipper-cast gel). Along with limited weight and size (10 kg, 162 × 412 × 290 mm), the above features make the instrument ideal for field use and other on-site investigations. Agilent 2100 can also be easily used by low-expertise staff. A similar system was previously employed to study the genetic variability of *bclA *gene for strain discrimination within the *Bacillus cereus *group [14]. In this paper we evaluate this approach for genotyping analysis of the two major biothreat agents.

## Results

### Experimental design

**Figure 1:**
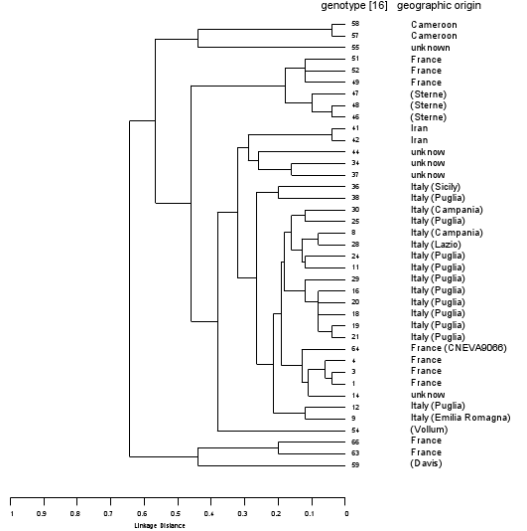
UPGMA dendrogram showing clustering and linkage distances of *B. anthracis *strains used to validate the Bioanalyzer 25 MLVA genotyping. The results obtained by previously described method [16] and Bioanalyzer genotyping are identical.

**Figure 2:**
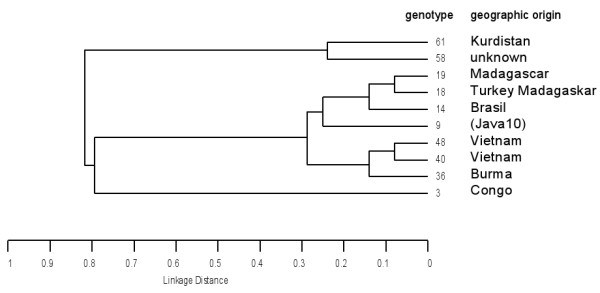
UPGMA dendrogram showing clustering and linkage distances of *Y. pestis *strains used to validate Agilent Bioanalyzer 2100 based genotyping. The results obtained by previously described method [15] and Bioanalyzer genotyping are identical.

In order to validate this platform, we compared the data produced by the Agilent 2100 Bioanalyzer to agarose gel based data (*B. anthracis*, *Y. pestis*) and capillary gel based data (*B. anthracis*). Ten *Y. pestis *and thirty-nine *B. anthracis *strains were genotyped using previously described sets of 25 VNTR loci [15,16]. These previously genotyped strains [15,16] were chosen to be representative of allelic variability within the two species. Briefly, these genotypes are from strains with the largest genetic distance, encompassing most of the observed allele variation (fig. 1 and 2). The 25 VNTR markers amplified for bacterial genotyping, were arranged into 12 PCR multiplex reactions either for *B. anthracis *or *Y. pestis*. This allowed us to use a single DNA 1000 chip for each strain.

After PCR amplification 1 μl of each reaction was loaded into 12-wells chip (DNA 1000 LabChip Kit). Primer concentrations were experimentally adjusted to obtain a balanced amplification of different fragments. This optimization was facilitated using the quantification data supplied by the 2100 Bioanalyzer (peak area and fluorescence peak level). The vast majority of data produced by Agilent 2100 were not accurate. The discrepancies between observed and expected fragment size (offset in Table 1 and 2) are, presumably, the result of abnormal migration patterns of some repetitive sequences into electrophoretic matrix. In order to check if the offset was reproducible, we tested interchip and intrachip variability in markers containing the smallest repeat units, for which incorrect allelic calling is more probable. The samples were run in triplicate on the same and on different chips, and size data compared. In general, it was observed a low level of inter-intrachip variability (less than 25 % of the repeat unit length). For example, for *B. anthracis *CG3 marker (UL = 5, so 25% UL = 1,25), we observed 168 ± 1 for allele 1 and 173 ± 1 for allele 2. To correctly convert the Agilent DNA fragment size estimates into repeat copy numbers, it was necessary to establish conversion tables (Table 1 and 2) containing 2100 Bioanalyzer fragment sizes as well as actual sizes corresponding to repeats unit numbers, as previously reported [16].

**Table 1:** Comparison between *B. anthracis *product sizes inferred by Agilent 2100 Bioanalyzer software (OBSERVED SIZE) and actual sizes obtained by direct sequencing of the PCR product or data available in Genbank (EXPECTED SIZE). UL BPS (unit length size).

**MULTIPLEX**	**MARKER (final primer concentration, μM)**	**UL BPS**	**ALLELE**	**EXPECTED SIZE**	**OBSERVED SIZE**	**OFFSET**
1	CG3 (1.1)	5	1	153	168	-15
			2	158	173	-15
	BAMS44 (0.8)	39	6	339	350	-11
			8	417	431	-14
	BAMS3 (0.6)	15	21	474	455	19
			24	519	499	20
			26	549	529	20
			27	564	546	18
			28	579	562	17
			30	609	586	23

2	vrrB2 (0.15)	9	4	135	141	-6
			6	153	159	-6
			7	162	168	-6
			8	171	177	-6
	BAMS5 (0.28)	39	3	229	230	-1
			5	307	297	10
			6	346	333	13
			7	385	370	15
			8	424	411	13
	BAMS15 (0.6)	9	24	418	427	-9
			27	445	448	-3
			41	571	581	-10
			42	580	595	-15
			44	598	613	-15
			45	607	619	-12
			46	616	624	-8

3	BAMS1 (0.6)	21	11	380	353	27
			12	401	371	30
			13	422	387	35
			14	443	404	39
			16	485	444	41
	vrrC1 (0.28)	9	46	517	501	16
			48	535	520	15
			53	583	564	19
			57	616	595	21

4	BAMS13 (0.28)	9	17	337	334	3
			20	364	362	2
			21	373	370	3
			24	391	386	5
			27	427	421	6
			30	454	452	2
			31	463	459	4
			47	607	597	10
			76	868	807	61

5	vrrB1 (0.15)	9	12	193	198	-5
			15	220	229	-9
			16	229	237	-8
			19	256	264	-8
	BAMS28 (0.6)	24	10	397	394	3
			13	469	468	1
			14	493	492	1
	vrrC2 (0.6)	18	17	532	515	17
			19	568	549	19
			21	604	583	21

6	BAMS53 (1.1)	12	6	212	224	-12
			8	236	249	-13
	BAMS31 (0.6)	9	15	331	332	-1
			49	637	638	-1
			55	691	674	17
			56	700	681	19
			57	709	684	25
			64	772	733	39
			65	781	742	39
			84	952	935	17

7	vrrA (0.1)	12	8	290	293	-3
			9	302	305	-3
			10	314	320	-6
			11	326	333	-7
	BAMS25 (1.1)	15	12	376	370	6
			13	391	381	10
	BAMS21 (1.1)	45	9	631	599	32
			10	676	629	47

8	BAMS34 (0.4)	39	8	425	399	26
			9	503	477	26
			11	581	550	31

9	BAMS24 (0.4)	42	9	511	526	-15
			10	553	571	-18
			11	595	608	-13

10	pXO1 (0.6)	3	6	123	135	-12
			7	126	138	-12
			8	129	141	-12
			9	132	145	-13
	BAMS51 (0.4)	45	6	358	338	20
			8	448	414	34
			9	493	468	25
			10	538	508	30
	BAMS22 (0.4)	36	11	555	527	28
			13	627	581	46
			14	663	624	39
			15	699	643	56
			16	735	659	76

11	BAMS23 (1.1)	42	5	399	377	22
			9	567	518	49
			10	609	557	52
			11	651	572	79
	pXO2 (0.28)	2	6	135	165	-30
			7	137	168	-31
			8	139	170	-31
			9	141	172	-31

12	BAMS30 (0.8)	9	6	268	269	-1
			17	367	360	7
			51	673	670	3
			57	727	685	42
			60	754	700	54
			68	826	770	56
			69	835	776	59
			72	862	796	66
			75	889	817	72
			78	916	840	76

**Table 2:** Comparison between *Y. pestis *product sizes inferred by Agilent 2100 Bioanalyzer software (OBSERVED SIZE) and actual sizes obtained by direct sequencing of the PCR product or data available in Genbank (EXPECTED SIZE). UL BPS (unit length size).

**MULTIPLEX**	**MARKER (final primer concentration, μM)**	**UL BPS**	**ALLELE**	**EXPECTED SIZE**	**OBSERVED SIZE**	**OFFSET**
1	ypms51 (0.2)	21	1	186	185	1
			2	207	207	0
	ypms01* (0.2)	18	6	283	279	4
			7	301	291	10
			9	337	329	8
			10	355	344	11

2	ypms04 (0.2)	17	5	179	187	-8
			6	196	205	-9
			8	230	241	-11
			9	247	259	-12
	ypms62* (0.2)	9	5	270	258	12
			6	279	267	12
			9	306	285	21
			10	315	294	21
			12	333	308	25
			15	360	331	29
			20	405	366	39

3	ypms07 (0.2)	10	7	164	168	-4
			8	174	178	-4
			9	184	187	-3
	ypms05 (0.2)	17	10	274	285	-11
			11	291	300	-9

4	ypms74 (0.2)	15	5	180	178	2
			6	195	190	5
			7	210	203	7
			8	225	217	8
	ypms15* (0.2)	15	9	275	272	3
			10	290	284	6

5	ypms35 (0.2)	15	8	204	208	-4
			9	219	222	-3
	ypms40* (0.2)	17	7	253	252	1
			8	270	266	4
			9	287	277	10

6	ypms38 (0.2)	16	6	201	200	1
			7	217	215	2
			8	233	230	3
	ypms20* (0.2)	15	8	288	285	3
			9	303	300	3

7	ypms41 (0.2)	17	5	183	188	-5
			6	200	207	-7
			7	217	221	-4
	ypms56* (0.2)	16	7	258	265	-7
			9	290	296	-6

8	ypms45 (0.2)	12	6	149	147	2
			7	161	159	2
	ypms44 (0.2)	17	7	233	239	-6
			8	250	260	-10
	ypms09* (0.2)	18	22	732	671	61
			32	930	847	83
			35	984	912	72

9	ypms71 (0.2)	14	5	157	163	-6
			6	171	178	-7
	ypms46 (0.2)	7	3	238	243	-5
			4	245	250	-5
			5	252	256	-4
			6	259	263	-4
			10	308	313	-5
			12	322	326	-4

10	ypms70 (0.2)	9	5	137	145	-8
			6	146	155	-9
			8	164	174	-10
			9	173	185	-12
			11	191	205	-14
	ypms54 (0.2)	22	6	259	265	-6
			7	281	289	-8

11	ypms69 (0.2)	16	5	163	164	-1
			6	179	177	2
			7	195	195	0
	ypms06 (0.2)	60	3	306	309	-3
			4	366	374	-8
			6	486	504	-18
			8	606	625	-19
			9	666	674	-8

12	ypms73 (0.2)	18	5	207	212	-5
			6	225	231	-6
	ypms21* (0.2)	18	9	329	339	-10
			10	347	358	-11
			11	365	375	-10

* modified primer set (increased final fragment size)

Once these reproducible offset values are taken into account, the MLVA assay as run on the Agilent exhibited concordant results with previous typing results for *B. anthracis *and *Y. pestis *[15,16]. We were able to resolve closely sized DNA fragments, and the analyzed samples showed concordance either with interchip repetitions or with expected size data. These data, used for UPGMA cluster analysis, generate dendrograms with linkage distance perfectly identical to those previously obtained by standard methodologies (Fig. 1 and 2). For each individual strain the Agilent assay is completed in about 30 minutes after PCR amplification. For a single strain to be genotyped, this time is significantly lower than 3 hours described in previous assay [16].

### Genotyping *B. anthracis *with the 25-loci MLVA

To fit all the 25 loci into a 12-wells chip the *B. anthracis *loci were combined into 5 triplex, 3 duplex and 4 singleplex (Table 1) PCR amplifications. The arrangement of different loci in the same multiplex is such as to avoid overlapping of VNTR markers size ranges. For four *B. anthracis *loci multiplexing was impossible, because of the large allele size range.

**Figure 3:**
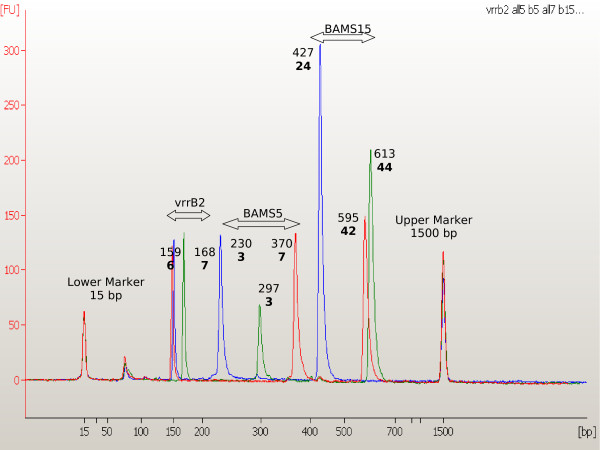
Multiplex n. 2 electropherogram comparison between three different *B. anthracis *strains (depicted in red, blue and green). For every amplicon the observed size and, in bold, the corresponding allele enumeration of markers vrrB2 BAMS5, and BAMS15 are shown.

Primer sequences were as described in [16], loci combination and final primers concentrations are reported in Table 1. Under these conditions, we were able to amplify each expected fragment, and the software (Agilent 2100 Expert version B.02.03.SI307, firmware C.01.055) could identify amplicon size (Fig. 3). The offset values for all loci and alleles were measured and are presented in Tables 1 and 2. For chromosomal markers, reproducibility of the observed data allowed correct assignment of each allele for every locus (<25% of unit length). However, for plasmid markers, since their shorter repeat unit and PCR product size compared to the chromosomal ones, we observed variability not exceeding one single additional base for each allele in repeated runs. Even with this additional base, correct assignment could be reproducibly done for the plasmid loci.

### Genotyping *Y. pestis *with the 25-loci MLVA

To fit all the 25 loci into 12 chip wells the *Y. pestis *25 markers were grouped into 11 duplex and one triplex (Table 2).

**Figure 4:**
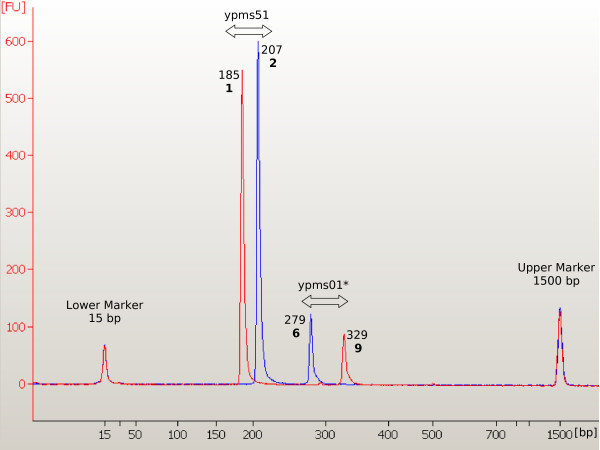
Multiplex n. 1 electropherogram comparison between two different *Y. pestis *strains (depicted in red and blue). For every amplicon the observed size and, in bold, the corresponding allele enumeration of markers ypms51 and ypms01 are shown. Yms01 is a locus for which modified primers pair is used.

The majority of primer sequences were as described in [15] but for 8 *Y. pestis *markers new primer pairs were redesigned to allow multiplexing (Table 3). Thus, final amplicon size was increased to avoid any possible overlap with other different loci into the new multiplexed reactions (Fig. 4). For *Y. pestis *markers (for which repeat units are at least 7 bp long [15,17]), we observed variability up to two bases (at the shortest repeat units) and, as previously described for *B. anthracis*, discrepancies not exceeding 25% of the repeat unit size.

**Table 3:** Modified primers for *Y. pestis *from [15] designed for the new multiplexed reactions.

**YP MODIFIED MARKERS**	**PRIMER SEQUENCE**
YPms01	F: ACTTCGATGATTATTTTGTGCGTA R: TGTTTGGTGCTATTGCCGTA
YPms09	F: TTTTTGCCGCACTGAACATA R: TCTGATGATTGTTGTGGTCGT
YPms15	F: ACAAGCAAGTTGCGCAGATA R: CAAGTTCGCTTTCTGTGTCG
YPms20	F: GCCAGAATAATGGCCGTAAA R: GTGGTTGTTCTTCACGTTGC
Ypms21	F: ACCGGCTTAAAGCAGATTGA R: TTCCCTGTACTCGATTGTTGTG
YPms40	F: TCCTGCTGCTGAGTTCATCTT R: TCATGTGCAATAGGCGTTGT
YPms56	F: GCTCTAACAACGCCGGTAAA R: ATGGCATCAACCGACTGACT
YPms62	F: AAACGGTCGTTAACGGAAGA R: GCTGAACAGCCCCATAAAAC

## Discussion

The intentional release of anthrax spores by mail in 2001 in the United States caused the death of five persons by inhalation anthrax and publicly demonstrated the bioweapon-associated threat, of which only the community of biodefence experts was previously aware. This increased the demands for a better genetic characterization of bioterrorism agents, in order to distinguish between natural outbreaks and/or intentional release of micro-organisms, and to help trace back the origin of an aggression. We have here evaluated the Agilent 2100 "Lab on a Chip" platform for genotyping of *B. anthracis *and *Y. pestis *strains on. This assay runs in 30 minutes one 12 multiplexed PCR reactions chip, genotyping a single isolate at a time. For this reason this system is faster compared to automated sequencing devices, either slab gel or capillary based when a single strain has to be genotyped. Given the possibility to compare different chip runs in different times this device can immediately show identities or diversities between new isolates compared to already characterized ones. Moreover it has shown a high degree of automation either for amplicon separation or for digital output of results. Compared to previous genotyping methods, the Bionalyzer is more effective than standard ethidium bromide slab gel electrophoresis, giving reproducible, precise and more sensible output for the shortest repeat units. Since no fluorescent primers are required the assay is cheaper to maintain than CEQ8000 or other sequencing machines. A rough estimation of the total cost of a 25 loci assay indicates that genotyping on this equipment is at least four to ten times less expensive than a capillary system assay, at low to medium throughput level. The equipment itself is cheaper, and both consumables and equipment can be stored for a long period of time and activated when needed.

Finally, the results are comparable to those obtained by automated sequencers. This platform appears particularly useful when response time is the critical factor. We propose therefore such a system as a method suitable for high resolution identification of biothreat agents. To date this system is the most effective genotyping technology available for on-site investigations. Also, this platform may be used for fast quality-checking of type collection, and DNA preparations for biosecurity and strain accountability purposes. Typing data produced by the MLVA approach can be easily compared using shared internet databases, as illustrated for instance in [18].

## Conclusion

In this paper we describe a fieldable genotyping method for *B. anthracis and Y. pestis*. This method is an adaptation on Agilent 2100 Bioanalyzer of previously described 25 loci MLVA. The system was validated by characterizing thirty-nine *B. anthracis *and ten *Y. pestis *isolates, demonstrating to be, for a single genotyping, more rapid than traditional methods. The transfer on a new platform maintains reproducibility and precision to unambigously identify alleles. This method is shown to be a valid alternative to standard genotyping techniques for field characterization of important biothreat agents.

## Methods

### Bacterial strains and isolates

The strains and DNA samples genotyped for this report are from the collection maintained by the French Ministry of Defence at Centre d'Etude du Bouchet (CEB), from Istituto Superiore di Sanità (ISS) Italian collections and from the Italian Reference Center for Anthrax (ISZ, Foggia) [16].

### VNTR amplification and analysis

DNA purification was performed as reported elsewhere [15,19]. Up to 30 ng of genomic DNA for each PCR reaction were used for DNA amplification in a final volume of 15 μl containing: 1× PCR reaction buffer (10 mM Tris-HCl, 1,5 mM MgCl_2_, 50 mM Kcl pH 8.3), 0.2 mM dNTPs, 1 U Taq polymerase and the appropriate concentrations of each primer as reported in Marker columns Table 1 and 2. PCR amplifications were conducted on a Peltier Thermal Cycler DNA Engine DYAD (MJ Research) as follow: initial denaturation step at 96°C for 3 min, 36 cycles of denaturation at 95°C for 20 seconds, annealing at 60°C for 30 seconds and extension at 72°C for 2 min. The reactions were terminated by a final incubation at 72°C for 5 min. Each reaction was loaded into chip wells prepared according to manufacturer recommendations (DNA 1000 LabChip Kit). Each chip contains 16 wells, 12 for the samples, 3 for gel mix (1 labeled black **G **and 2 grey **G**) and 1 for the ladder. Briefly, the black **G **well of DNA LabChip support was filled with 9 μl of a gel containing intercalating dye. The DNA LabChip was pressurized by a syringe in a priming station device and released after 60 sec. Then, two grey **G **wells were filled with 9 μl of gel-dye mix. After gel preparation, each sample well was loaded with 1 μl of PCR reaction and 5 μl of internal marker (containing two MW size standards of 15 and 1500 bp). Finally 1 μl of DNA ladder was loaded in the ladder well, the chip vortexed for 60 sec and inserted into Agilent 2100 Bioanalyzer. During the run the instrument analyzed sequentially every sample, showing electropherogram, virtual gel image and table data.

## Authors' contributions

GF, SG and ACia did the set up of the *B. anthracis *25-MLVA assay. VP, SG and ACia did the set up of the Y.*pestis *25-MLVA assay. ACia, RDes and SG participated to typing work. FL, ACas and GV did the error checking analysis. VP, ACia, and ACar did various sequence analysis. RD'am and ACas did error checking of overall sequence analysis. GV was in charge of the Bionumerics database and clustering analyses. FL, ACia, RD'am and GV conceived the study. FL and GV wrote the report. All authors read and approved the final manuscript.
